# Supplementary figures and images for: Genome-Wide Identification, Characterization, and Expression Analysis of Tubby-like Protein (TLP) Gene Family Members in Woodland Strawberry (Fragaria vesca)
Source: Int J Mol Sci. 2022 Oct 8;23(19):11961. doi: 10.3390/ijms231911961 (PMC9569999; doi:10.3390/ijms231911961)

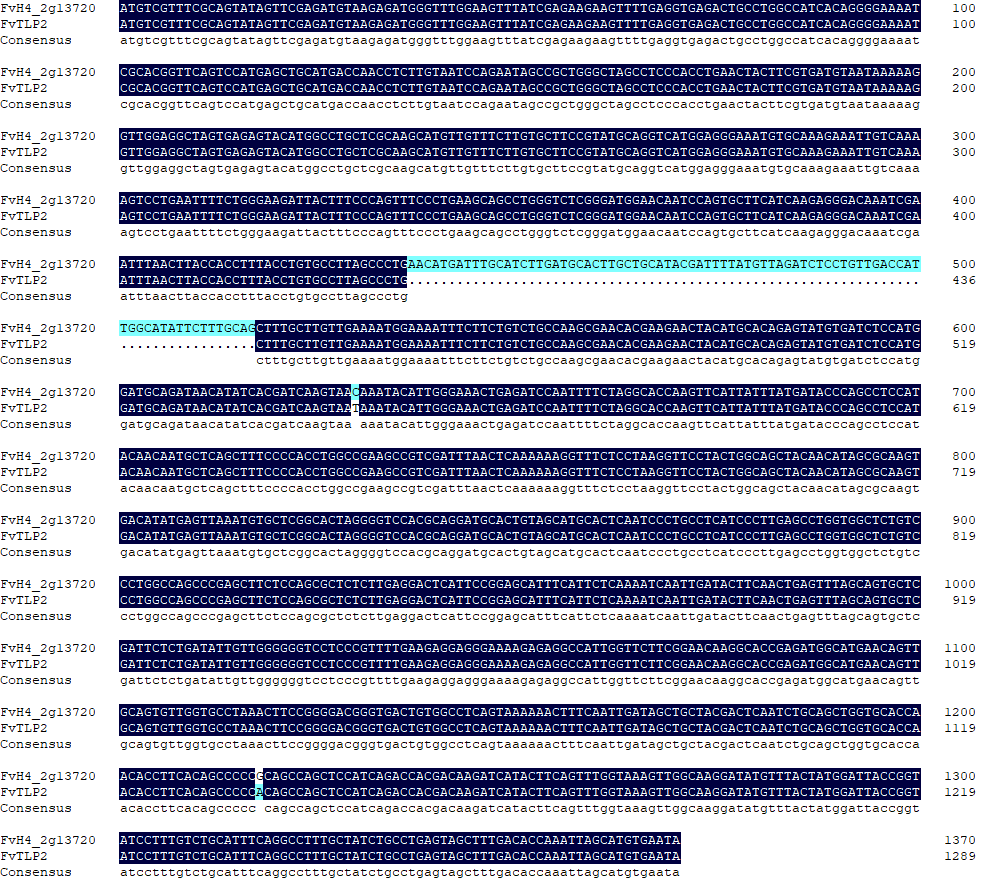

Supplement: Supplementary file 1 [file ijms-23-11961-s001.zip › Figure S1.tif]

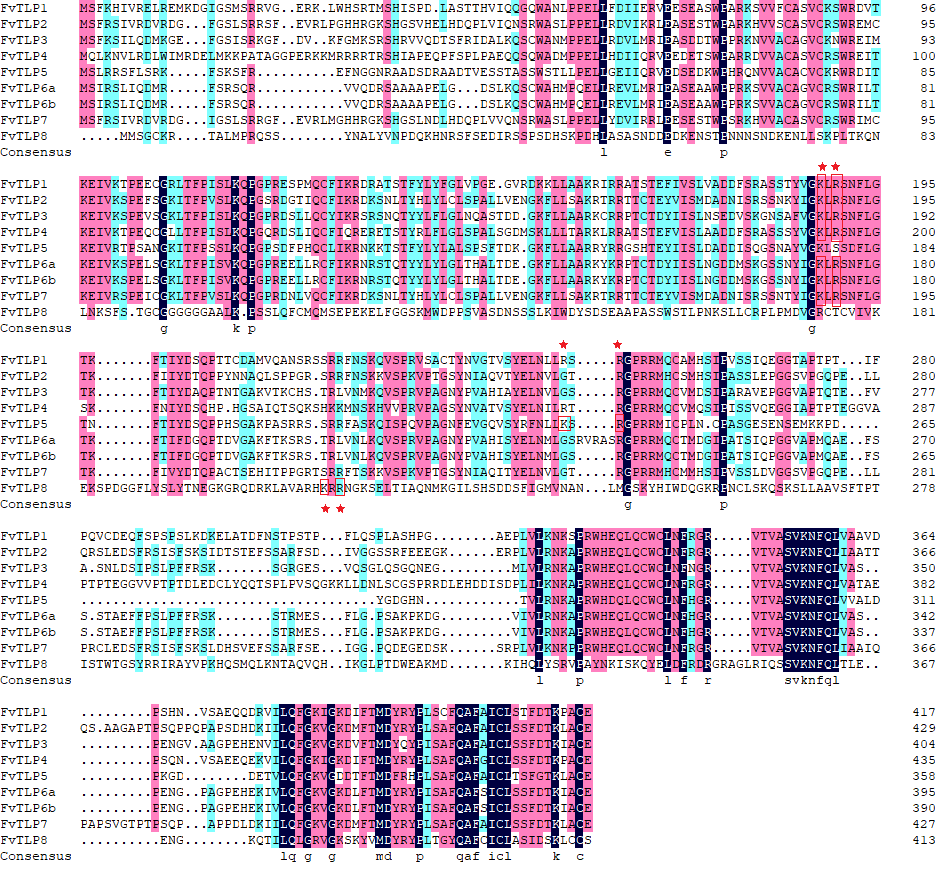

Supplement: Supplementary file 1 [file ijms-23-11961-s001.zip › Figure S2.tif]

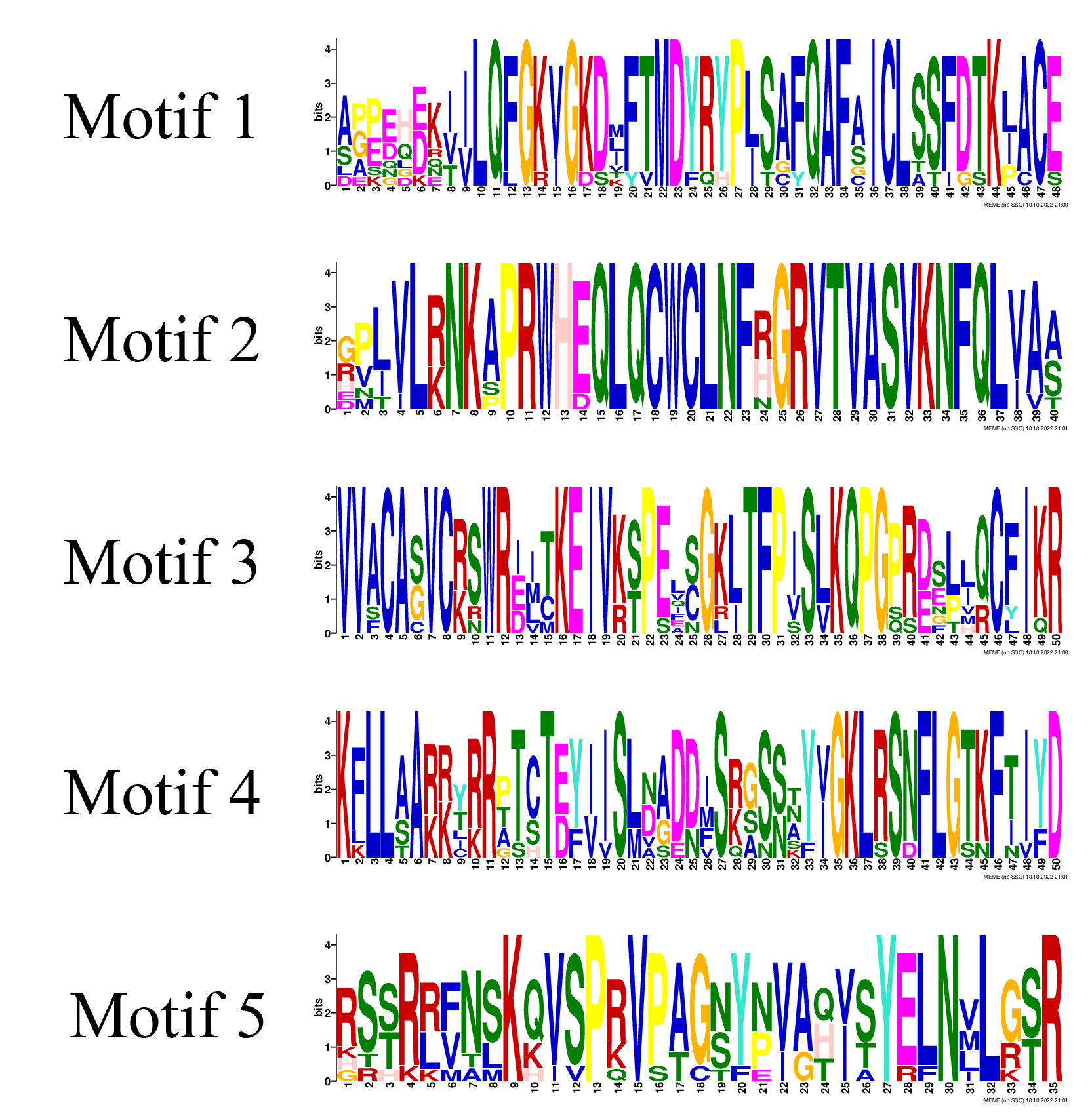

Supplement: Supplementary file 1 [file ijms-23-11961-s001.zip › Figure S3.tif]

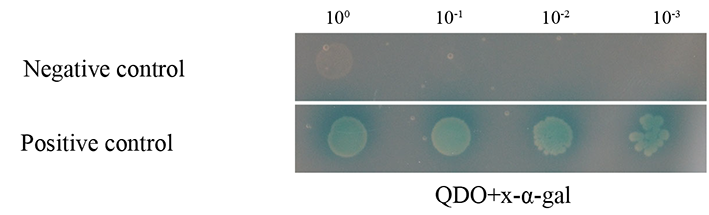

Supplement: Supplementary file 1 [file ijms-23-11961-s001.zip › Figure S4.tif]

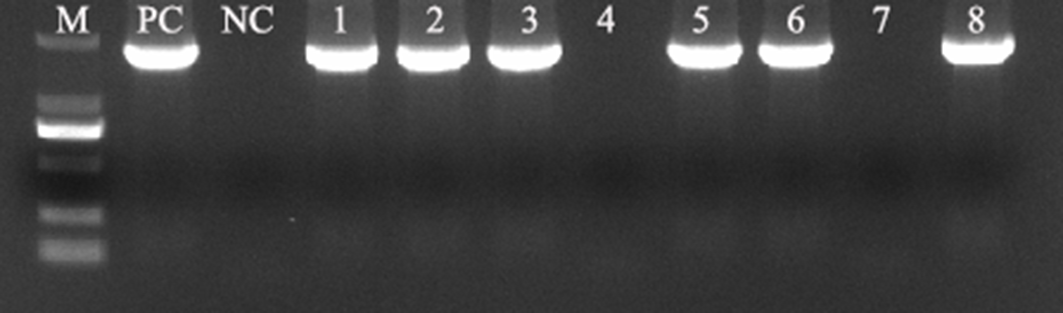

Supplement: Supplementary file 1 [file ijms-23-11961-s001.zip › Figure S5.tif]
